# Cancer Immunology and Cancer Immunodiagnosis 2016

**DOI:** 10.1155/2017/9127382

**Published:** 2017-01-10

**Authors:** Jianying Zhang, Bin Zhang, Yi Zhang

**Affiliations:** ^1^Cancer Autoimmunity Research Laboratory, Department of Biological Sciences, University of Texas at El Paso, El Paso, TX, USA; ^2^Division of Hematology and Oncology, Northwestern University Feinberg School of Medicine, Chicago, IL, USA; ^3^Biotherapy Center and Department of Oncology, The First Affiliated Hospital, Zhengzhou University, Zhengzhou, China

Cancer immunology is a branch of immunology that studies interactions between the immune system and cancer cells, which is a growing field of research that aims to identify biomarkers in cancer immunodiagnosis and to discover innovative cancer immunotherapies. The immune response, including the identification and recognition of cancer-specific antigens, is of particular interest in cancer immunology field as knowledge gained drives the development of new vaccines and antibody therapies. Activation of the immune system for therapeutic benefit against cancer has long been a goal in immunooncology. The passive cancer immunotherapy has been well-established for several decades, and continued advances in antibody and T-cell engineering should further enhance their clinical impact in the years to come. In contrast to these passive immunotherapy strategies, the active cancer immunotherapy has been proved elusive. In the context of advances in the understanding of how tolerance, immunity, and immunosuppression regulate antitumor immune responses together with the advent of targeted therapies, these successes suggest that active immunotherapy represents a path to obtain a durable and long-lasting response in cancer patients. The key to cancer immunodiagnosis and immunotherapy is an improved understanding of the immune response during cancer initiation and progression.

According to this background, we have invited investigators to contribute original research articles as well as review articles describing cancer immunodiagnosis and cancer immunotherapy (CICI) and assembled this special issue for updating the recent advances in this field. In this special CICI issue, we have received 23 submitted manuscripts, and 8 manuscripts have been accepted for publication and included in this special issue. For example, a paper of V. Kaewkangsadan et al. has characterized the contribution of T effector/regulatory cells and cytokines to tumor cell death with neoadjuvant chemotherapy (NAC); a paper of L. Qian et al. demonstrated that adoptive cellular immunotherapy (CIT) contributes to improvement of prognosis and inhibition of viral replication in HCV-related HCC patients, without impairment of liver function; a paper of Z. Lu et al. has indicated that p-p70S6K may participate in the invasion and metastasis in the development of esophageal squamous cell carcinoma (ESCC) and downregulation of the expression of p-p70S6K could improve the sensitivity of cells to rapamycin in ESCC; a paper of M. M. Lotem et al. has discussed the adjuvant autologous melanoma vaccine for macroscopic stage III disease. In addition, four review manuscripts were also included in this special issue.

In summary, this special issue covers many important aspects in cancer immunology, including recent advances in the basic and clinical studies relating to cancer immunotherapy. We hope that this special issue can provide valuable information to investigators in the field of CICI and also give the readers a sense of some of the advancements made in this field.

*Jianying Zhang* *Bin Zhang* *Yi Zhang*

